# Determining the effectiveness of a video-based contact intervention in improving attitudes of Penang primary care nurses towards people with mental illness

**DOI:** 10.1371/journal.pone.0187861

**Published:** 2017-11-13

**Authors:** Yin Ping Ng, Abdul Rashid, Finian O’Brien

**Affiliations:** 1 Department of Psychiatry, Penang Medical College, Penang, Malaysia; 2 Department of Public Health, Penang Medical College, Penang, Malaysia; University of West London, UNITED KINGDOM

## Abstract

**Background:**

Mental illness-related stigma is common, and is associated with poorer outcomes in people with mental illness. This study evaluated the attitudes of primary care nurses towards people with mental illness and its associated factors; and the effectiveness of a short video-based contact intervention (VBCI) in improving these attitudes using a Malay version of the 15-item Opening Minds Stigma Scale for Healthcare Providers (OMS-HC-15-M).

**Methods:**

A 5-minute VBCI was developed comprising elements of psychoeducation and interviews of people with mental illness and the people they interact with, relating to experience of mental illness and recovery. A pre-post cross-sectional study was conducted on 206 randomly selected primary care nurses in Penang, Malaysia. The OMS-HC-15-M questionnaire was administered before and immediately after participants viewed the VBCI. The difference in mean pre-post VBCI scores using paired t-tests, effect size and standardised response mean (SRM) were obtained. Factors correlating to attitudes were obtained using univariate and multivariate regression analyses.

**Results:**

Differences in pre-post VBCI score were statistically significant (p<0.001) with a 14% score reduction, a moderate effect size and SRM at 0.97 (0.85–0.11) and 1.1 (0.97–1.2) respectively. By factoring in the Minimal Detectable Change statistic of 7.76, the VBCI produced a significant improvement of attitudes in 30% of the participants. Factors associated with less stigmatising attitudes at baseline were previous psychiatry-related training, desiring psychiatric training, and positive contact with people with mental illness.

**Conclusions:**

This is the first study in Malaysia to show that a brief VBCI is effective in improving attitudes of primary care nurses towards people with mental illness in the immediate term. Further studies are needed to determine if these results can be sustained in the longer term and generalizable to other health care professionals. Qualitative studies are warranted to provide insight to the factors correlating to these attitudes. (300 words)

## Introduction

Mental illness-related stigma was defined by Goffman (1963) as a ‘discrediting trait’ which results in negative attitudes towards people with mental illness [1, 2]. Attitudes towards people with mental illness are influenced by many factors, including culture, level of education, knowledge, and contact with people with mental illness [3–20]. Culture shapes family beliefs, media portrayals of people with mental illness and local health care provisions for mental health [4, 6]. For example, mental illness is often perceived in many cultures as a sign of possession by evil spirits, moral weakness, a curse or a form of punishment by higher beings [7, 16, 17, 21]. Lower mental health literacy has been linked to more stigmatising attitudes while greater mental health literacy is associated with improved attitudes towards people with mental illness [8–11, 18–20]. Most studies have found contact with people with mental illness to be associated to lower levels of stigma [10, 12–15, 20], while others did not find such an association [22, 23].

Mental illness-related stigma has been shown to lead to social withdrawal, self-stigma, help-seeking avoidance, and overall poorer outcomes in people with mental illness [24, 25]. It is a key deterrent in mental health treatment and recovery efforts [25]. Unfortunately, stigmatising attitudes are not confined to the general community, but also prevalent among healthcare professionals (HCPs) [4, 26–28]. This is concerning because HCPs are responsible for providing care and health education to vulnerable members of society, and such prejudice may adversely affect quality of care rendered to people with mental illness. A review by Henderson and colleagues [27] outlines evidence of the negative effects of such prejudice on the quality of health care for people with mental illness.

There is a paucity of research on stigma amongst HCPs towards people with mental illness in Malaysia, so that, by time of writing, there were just three publications relating to this topic [29–31], of which two were qualitative studies. Ashencaen [29] found stigmatising attitudes among the psychiatric nursing staff in Sarawak state towards people with mental illness. Also, Hanafiah and Bortel [30] explored the perspectives of 15 Malaysian mental health professionals and found that these HCPs believed family members, friends and workplace staff, (particularly nurses) to be the greatest perpetrator of mental illness-related stigma. The study did not, however, explore the HCPS own attitudes towards people with mental illness. The sole quantitative study by Minas et al [31] found that Malaysian HCPs were more discriminatory towards people with mental illness compared to those with diabetes mellitus. However, the authors did not utilize a scale specifically designed for use with HCPs [29–31].

Previous research studies have utilised a number of different scales to measure the attitudes of HCPs towards people with mental illness [10, 31–36]. However, most of these have significant limitations in terms of applicability, in that either they are rather lengthy and impractical for use in busy clinic settings, do not have established psychometric validity, or were developed to assess attitudes of the general population towards people with mental rather than HCPs [36–39]. In particular, the use of scales that were not evaluated for use in HCPs raises questions about the validity of the results obtained using these instruments [36]. Moreover, some questions contained in these scales may be inappropriate or irrelevant in assessing prejudice among HCPs. For example, questions such as whether the respondent endorses the biomedical model relating to the causality of mental illness, or whether they believed that people who develop mental illness are themselves the cause of their health problems, may not be relevant questions in examining mental illness-related stigma among HCPs [36, 40, 41]. On the other hand, other components pertinent to HCPs are not addressed in these scales, such as whether a HCP would disclose that they have mental illness to others, or whether they had positive or negative attitudes towards psychiatry as a medical profession [36, 42].

A number of scales have been specifically developed to measure attitudes of HCPs towards mental illness, for example, the Attitudes Towards Acute Mental Health Scale (ATAMHS-33), the Mental Illness: Clinician Scale (MICA) and the Opening Minds Scale for Health Care Providers (OMS-HC). The ATAMHS-33 was designed to survey the attitudes of acute mental health staff [43], while the MICA was originally developed to measure attitudes of medical students towards people with mental illness, but later modified to be used in HCPs [36]. The OMS-HC is a newly-developed scale that evaluates attitudes of HCPs towards people with mental illness. This 15-item scale (OMS-HC-15) contains four questions adapted from the MICA [44, 45].

We identified 2 published reviews which evaluated anti-stigma interventions in HCPs [26, 27]. Both reviews noted that the interventions were largely educational, with some employing social contact. Social contact is defined as a planned interaction between the individual and person(s) with mental illness [46]. The reviews concluded that interventions incorporating social contact are more effective compared to other interventions in reducing stigmatising attitudes among HCPs in the short term [26, 27], with one review [26] noting interventions with filmed or video-based contact were as effective as face-to-face social contact. In their review, Henderson and colleagues [27] suggested that educational interventions may be effective in HCPs with little training in mental health. In addition, they also noted that only 2 studies were focused on mental disorders in general, while the others involved interventions aimed at improving attitudes towards people with specific mental health conditions such as substance use disorders and self-harm behaviour, which limited the applicability of these interventions to other mental disorders. By the time of writing, there are no published studies evaluating anti-stigma interventions among HCPs in Malaysia.

In summary, despite evidence of widespread stigma towards people with mental illness, and an internationally-recognised need for ongoing anti-stigma interventions in society, there is a paucity of research on mental illness-related stigma among HCPs in Malaysia. No previous Malaysian study utilised assessment scales specifically-designed for use with HCPs, or evaluated anti-stigma interventions with HCPs. As nurses are frontline HCPs who frequently encounter people with mental illness in the primary care sector, this study aimed to assess the attitudes of government-employed primary care nurses in Penang, Malaysia towards people with mental illness by using the OMS-HC-15 scale, and to determine whether the use of a simple and relatively inexpensive video-based contact intervention would be effective in reducing mental illness-related stigma in this group.

## Materials and methods

### Research design

This is a before and after, quasi-interventional study conducted from 1st April to 31st July 2016.

### Study setting

There are 26 government primary care clinics in Penang, each manned by allied health staff (including assistant nurses, community nurses, staff nurse, head nurses and matrons) and headed by either a medical officer or a Family Medicine Specialist. These clinics serve the local community and are often the location of first medical contact for people with mental illness. According to the recent Malaysian National Health and Morbidity Survey, nearly 30% of Malaysians aged 16 years and above suffer from mental health problems [47].

### Sample

At the time of this study, there were 1137 primary care nurses employed in the state of Penang. Participants were included if they were full time primary care nurses working in government clinics and sufficiently proficient in Malay to complete the study questionnaires. The sample size was calculated based on the pre- and post-intervention scores from the psychometric evaluation of the OMS-HC-15 [48], at 90% of power and 95% significance level. A smaller difference of 0.11 was used compared to that used in the study by Modgill and colleagues [48] to produce a larger sample size of 201 participants. When an estimated 20% drop-out was included, the resultant sample size was 242 participants.

Participants were selected from a list containing full time primary care nurses obtained from the State Health Department. Potential participants were listed from 1 to 1137; and 242 names were generated randomly from the list using the ‘Research Randomizer’ computer software. The nurses selected using this method were then invited to attend the study briefing and subsequently recruited if they wanted to participate, upon provision of written informed consent.

### Materials

#### The attitudes of nurses to people presenting with mental illness (ANPMI) study questionnaire

A proforma in the local Malay language was developed consisting of questions on demographic characteristics, experience with mental illness and Malay translation of the 15-item Opening Minds Scale for Health Care Providers Scale (OMS-HC-15).

The OMS-HC-15 is a self-report questionnaire developed by the Canadian Mental Health Commission to assess attitudes and behavioural intentions of HCPs specifically relating to 3 dimensions: negative attitudes, preference for social distance and willingness to disclose and seek help [48]. The OMS-HC-15 was chosen because it is specifically designed for use with HCPs, was freely available, and time-efficient in that it comprises just 15 items. In addition, the OMS-HC-15 has been psychometrically validated with an acceptable internal consistency (α = 0.79), construct validity, meaningful factorial structure and responsiveness to change. It has been used as a tool to measure effectiveness of anti-stigma intervention programs among different groups of HCPs and demonstrated moderate sensitivity to change [48]. Each item in the scale is scored on a Likert scale where 1 = Strongly disagree, 2 = Disagree, 3 = Neither agree nor disagree, 4 = Agree and 5 = Strongly agree. Items 2,6,7,8 and 14 require reverse coding. The sum of all items provided a total score ranging from 15 to 75. Higher scores imply a more stigmatizing attitude [48].

To cater to the predominantly Malay speaking nurses, a modified Malay version of the OMS-HC-15 was used in this study. Permission to utilize and translate the questionnaire was obtained from the original author(s). The Malay version of the OMS-HC-15 (OMS-HC-15-M) was created via a procedure of forward translation into Malay and a blind back-translation into English [49], after which the back-translated English version was assessed for face validity, and semantic and conceptual equivalence. The internal consistency of the OMS-HC-15-M was acceptable with a Cronbach alpha of 0.76. For this study, the OMS-HC-15-M was administered at 2 time points: time point 1 (T1), prior to the intervention and time point 2 (T2) immediately after the intervention.

#### Video-based contact intervention (VBCI)

(available on https://www.youtube.com/watch?v=K2cOe_dVUng&t=16s)

Knaak and Patten [2] recommended inclusion of six key ingredients in anti-stigma interventions to maximise their effectiveness. These comprise: (i) social contact in a form of personal testimony by trained speaker(s) with lived experience of mental illness, (ii) utilization of multiple points of social contact, (iii) skills-training for HCPs focusing on behaviour change, (iv) myth busting, (v) utilization of passionate facilitators who model a person-centred approach, and finally, (vi) emphasis in recovery as a key message [2]. A video-based medium was chosen because it is less labour intensive, inexpensive, easy to reproduce and has the potential of reaching a broader audience compared to direct face-to-face contact intervention. Based on the recommendations [2], a VBCI was constructed by the researchers consisting of:

1. An anti-stigma awareness video [50] which depicted an accident victim treated with stereotyped negative attitudes towards people with mental illness;
2. Common myths regarding people with mental illness
3. A video clip featuring celebrities with mental illness
4. Filmed testimonies by a patient with mental illness and her visiting community nurse; focusing on the patient’s life, struggles and perseverance against stigma [51]
5. An interview with a successful person with mental illness in recovery [52]
6. A personal testimony of a layperson on her colleague with mental illness (in recovery and gainfully working) with emphasis that despite having mental illness, the colleague was the same any normal person [53]; and finally
7. Mental health facts to correct myths, disconfirm stereotypes and promote mental health awareness; with basic tips on ways to help people with mental illness

During the video development, the first author sought expert opinion on the video content from the authors of the key recommendations [2]. Four video clips were adapted from Youtube videos that were freely available on the internet, and due acknowledgement was given for each clip at the end of the video presentation [50–53]. It was not feasible to create original video footages due to time and financial constraints. The video was produced by the first author, aided by technical expertise provided by an audio-visual production company, Brilliant Entertainment Studios. The video was shown to 10 HCPs before and after revision and deemed satisfactory in terms of the audio and picture quality, subtitling and language.

### Study process

Nurses who consented to participate completed the study questionnaire, which was then sealed in opaque envelopes and collected at T1. These envelopes were identified only by the participants’ unique identification numbers (ID) to maintain confidentiality. The VBCI was then shown to the participants. Prior to that, the participants were informed not to divulge their opinions regarding the intervention until the end of the study. No additional information was given regarding the video to minimize potential bias. Immediately after the VBCI, the nurses completed a second set of the OMS-HC-15-M, which was collected in sealed opaque envelopes at T2.

### Analysis

The data was analysed using STATA 14.0. Demographic characteristics were analysed using descriptive statistics. The mean scores for total OMS-HC-15 and its subscales were obtained by totalling the raw scores divided by the number of individual items within the scale/ subscale. Testing for normality and homogeneity of variances using Shapiro-Wilk’s test and Levene’s test were done, followed by univariate (t-tests and one-way ANOVA) and multivariate analyses to determine the factors associated with mean total OMS-HC-15-M scores at baseline.

The effectiveness of the VBCI was determined via (i) the OMS-HC-15-M change scores using paired t-tests; (ii) effect size [54, 55]; and (iii) standardized response mean (SRM) [56, 57]. The effect size was measured by using the formulae ‘effect size = mean change in scores over standard deviation of the pre-intervention mean score’ [54, 58] while the SRM was calculated using the change in scores divided by the standard deviation of the change scores [56, 57]. The effect of time on the change scores was evaluated using the general linear model repeated measures test. The effect size was interpreted using Hopkin’s Likert scale approach; where the effect size of 0 - <0.2 = trivial effect, 0.2 - <0.6 = small effect, 0.6 - <1.2 = moderate effect, 1.2 - < 2 = large effect, 2 - <4 = very large effect and 4 –infinity being considered as ‘nearly perfect’ [54, 55].

To examine the impact of the VBCI in reducing stigma, the minimum detectable change (MDC) statistic was factored into the change scores, represented by the formula: [z score * √2 * standard error measurement (SEM)]. The MDC reflects the smallest difference or true change that can be detected which is not due to chance or systematic error [59]. For this study, the z score of the 95% confidence interval, 1.96; and the SEM of 2.80 (derived from test-retest results of the full 20-item scale due to unavailability of the OMS-HC-15 test-retest results) was used to obtain a MDC of 7.76. Using the MDC, an increase or decrease of at least 7.76 points on the OMS-HC-15-M would reflect a true change in attitude.

### Ethical approval

The study was approved by the Malaysian Research Ethics Committee (Document Number: (13) KKM/NIHSEC/P16-263) and the Penang State Department Health Director, and was conducted according to the principles expressed in the ICH Harmonized Tripartite Guidelines for Good Clinical Practice and the Declaration of Helsinki.

## Results

### Response rate

**Fig 1:**
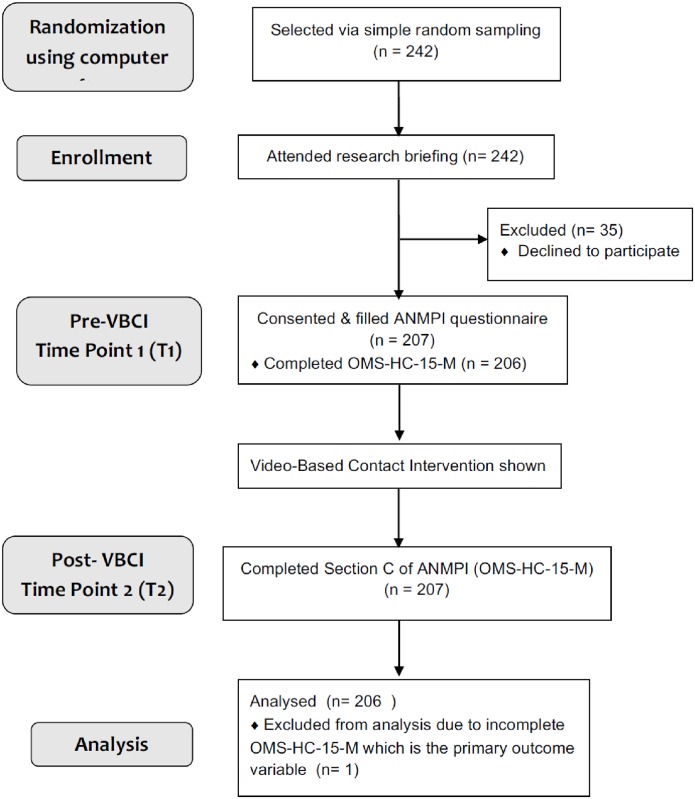
Study flow. ANMPI, The attitudes of nurses to people presenting with mental illness; OMS-HC-15-M, Opening Minds Scale for Healthcare Providers 15 Items, Malay version.

207 nurses participated in the study, resulting in a response rate of 86% of those who were invited to participate. There were no drop-outs. Participants were not required to provide reasons for not agreeing to participate. One participant did not complete the pre-intervention OMS-HC-15-M and was omitted from analysis. Fig 1 shows a diagram of the study flow.

### Baseline profile of the participants

Participant characteristics are summarised in Table 1. The mean age of participants was 33 (22–59) years, with 84% (n = 173) being 40 years and below. Only 3 respondents were male. The majority of nurses were Malay (93%, n = 191), Muslim (93%, n = 192), married (77%, n = 159) and had obtained either a certificate (49% (n = 93) or a diploma (49% (n = 93) as their highest undergraduate qualifications. Slightly more than half (55%, n = 113) worked as community nurses.

**Table 1:** Demographic characteristics of participants (n = 206).

Independent variables		Frequency	% (95% CI)
Age groups (n = 206)	21years– 40 years	173	84 (78–88)
	41year– 60 years	33	16 (12–22)
Gender (n = 206)	Female	203	99 (95–100)
	Male	3	1.5 (0.47–4.5)
Ethnicity (n = 206)	Malay	191	93 (88–96)
	Chinese	4	1.9 (0.72–5.1)
	Indian	11	5.3 (3.0–9.4)
Religion (n = 206)	Muslim	192	93 (89–96)
	Buddhist	3	1.5 (0.47–4.5)
	Christian	2	1.0 (0.24–3.8)
	Hindu	9	4.4 (2.3–8.2)
Marital status (n = 206)	Single	37	18 (13–24)
	Married	159	77 (71–82)
	Separated	1	0.49 (0.07–3.4)
	Divorced	5	2.4 (1.0–5.7)
	Widowed	4	1.9 (0.72–5.1)
Highest qualification (n = 190)	Certificate	93	49 (42–56)
	Diploma	93	49 (42–56)
	Degree	4	2.1 (7.8–5.5)
Rank (n = 206)	Community nurse	113	55 (48–62)
	Staff nurse	76	37 (31–44)
	Head nurse	10	4.9 (2.6–8.8)
	Matron	7	3.4 (1.6–7.0)

CI, confidence interval.

The respondents’ experiences with mental illness is summarised in Table 2. Only 1% (n = 2) reported they had ever received psychiatric treatment. Ninety percent (n = 186) had encountered people with mental illness; with approximately two-thirds reporting that they had contact with people with mental illness either at their workplace (n = 126), or outside their workplace (n = 122). The majority (68%, n = 127) reported that they encountered people with mental illness only rarely. The majority of the respondents (86%, n = 174) stated that they wanted (more) psychiatric training, while less than a third (n = 60) reported they had received any previous psychiatric training. Only 8% (n = 13) found their experience with people with mental illness negative while nearly equal numbers rated their experience as positive (45%, n = 75) or neutral (47%, n = 79).

**Table 2:** Experiences with mental illness.

Independent variables		Frequency	% (95% CI)
Received previous or current psychiatric treatment (n = 206)	No	204	99 (96–100)
	Yes	2	0.97 (0.24–3.8)
Had past contact with PWMI (n = 206)	No	20	9.7 (6.3–15)
	Yes	186	90 (85–94)
Had past contact with family or close friends with MI (n = 186)	No	168	90 (85–94)
	Yes	18	9.7 (6.2–15)
Had past contact with acquaintances with MI (n = 186)	No	173	93 (88–96)
	Yes	13	7.0 (4.1–12)
Had past contact with patients at workplace with MI (n = 186)	No	60	32 (26–39)
	Yes	126	68 (61–74)
Had past contact with PWMI outside workplace (n = 186)	No	64	34 (28–42)
	Yes	122	66 (58–72)
Nature of experience with PWMI (n = 167)	Positive	75	45 (37–53)
	Negative	13	7.8 (4.5–13)
	Neutral	79	47 (40–55)
Frequency of encounter (n = 186)	Daily	13	7.0 (4.1–11)
	At least once a week	21	11 (7.4–17)
	At least once a month	25	13 (9.2–19)
	Rarely	127	68 (61–75)
Received previous psychiatry training (n = 204)	No	144	71 (64–76)
	Yes	60	29 (24–36)
Desire psychiatric training (n = 203)	No	29	14(10–20)
	Yes	174	86 (80–90)

CI, confidence interval; PWMI, people with mental illness; MI, mental illness.

### OMS-HC-15-M mean scores at baseline, post-VBCI, and change scores

Table 3 shows the mean change scores for the total OMS-HC-15 scale and subscales. Overall, there were statistically significant improvements in the mean scores. The mean total scale score decreased by 14% while the Attitude, Disclosure and Help-seeking and Social Distance mean subscale scores increased by 14%, 11% and 18% respectively. Repeated measures testing was equally statistically significant for total scale (F = 251, *p* < 0.001), Attitude subscale (F = 155, *p* < 0.001), Disclosure and Help-seeking subscale (F = 58, *p* < 0.001) and Social Distance subscale (F = 170, *p* < 0.001).

**Table 3:** OMS-HC-15-M pre-post intervention change scores.

OMS-HC-15-M scores	Pre-VBCI (mean, 95% CI), n = 206	Post-VBCI (mean, 95% CI), n = 206	Difference (mean, 95% CI), n = 206	t test	*p* value
Total scale (15 items)	40 (39–40)	34 (33–35)	-5.6 (-6.2 - -4.9)	16	<0.001*
Attitude sub-scale (6 items)	16 (16–17)	14 (14–15)	-2.2 (-2.5 - -1.8)	-13	<0.001*
Help-seeking & Disclosure subscale (4 items)	9.8 (9.5–10)	8.7 (8.5–9.0)	-1.1(-1.4 - -0.81)	-7.6	<0.001*
Social Distance subscale (5 items)	13 (13–14)	11 (11–12)	-2.3 (-2.6 - -2.0)	-13	<0.001*

OMS-HC-15-M, Opening Minds Scale for Healthcare Providers– 15 Items Malay version; VBCI, Video-based Contact Intervention; CI, confidence interval.

*p<0.05.

### Factors correlating to mean total scores at baseline

From the univariate analyses (see tables in S1 & S2 Tables), independent variables found to be statistically significantly associated with lower mean total OMS-HC-15-M scores were previous contact with people with mental illness, previous psychiatry-related training, desiring psychiatric training, and nature of experience with people with mental illness. Following multivariate regression, factors which significantly correlated with lower OMS-HC-15-M scores at baseline were previous psychiatric training, desiring psychiatric training, and positive experienced with people with mental illness (F = 5.6, *p* < 0.001, R^2^ = 0.12). The results of the multivariate regression analysis are shown in Table 4.

**Table 4:** Multivariate regression analysis for independent variables associated with mean total pre-VBCI OMS-HC-15-M scores.

Pre-VBCI total OMS-HC-15-M scores (n = 164)	Coefficient	t (95% CI)	*p* value
Received previous training	-1.7	-2.0 (-3.4 - -0.01)	0.049*
Desire to be trained	-3.3	-2.4 (-5.9 - -0.74)	0.012*
Nature of experience with PWMI			
Negative	1.6	1.0 (-1.5–4.7)	0.310
Neutral	2.7	3.2 (1.0–4.4)	0.002*
Cons	41	31 (39–44)	0.001

OMS-HC-15-M, Opening Minds Scale for Healthcare Providers– 15 Items Malay version; PWMI, people with mental illness; CI, confidence interval

*p<0.05, F = 5.6, *p* < 0.001, R^2^ = 0.12

### Effect size, standardised response mean (SRM) and responsiveness of the OMS-HC-15-M

The effect size and standardised response mean were both moderate at 0.97 (0.85–1.1) and 1.1 (0.97–1.2) with narrow confidence intervals, which also demonstrated that the OMS-HC-15-M is responsive to positive change [57, 60].

### Impact of VBCI in reducing stigma

**Fig 2:**
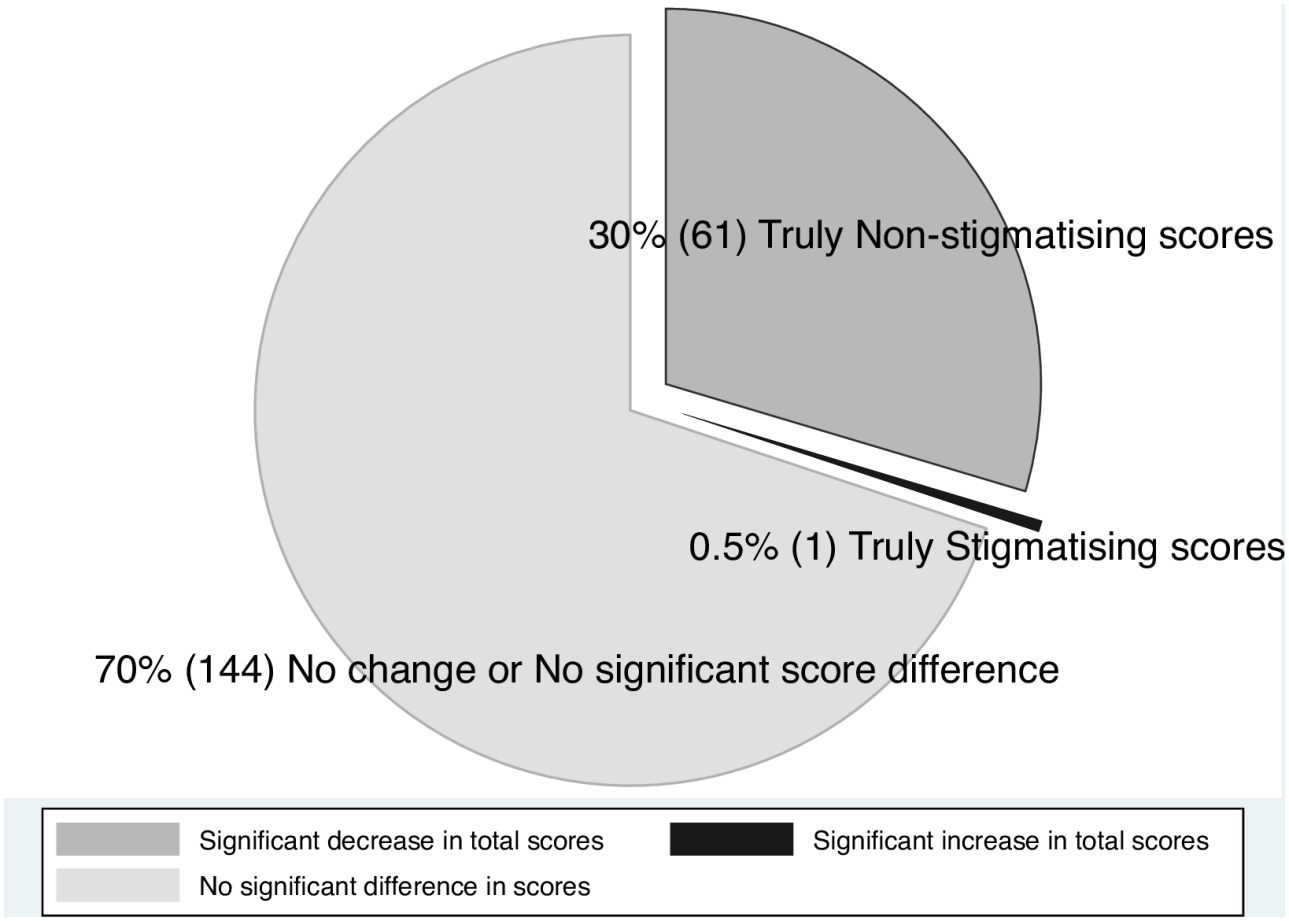
Change scores with minimal detectable change applied.

After incorporating the MDC, 30% (95% CI 24–36, n = 61) had reduced scores after the VBCI while only one nurse obtained a higher score (Fig 2).

## Discussion

This is the first Malaysian study to determine the effectiveness of a short video-based contact anti-stigma intervention amongst primary care HCPs in Malaysia, and to evaluate the attitudes of primary care nurses towards people with mental illness.

### Effectiveness of the VBCI as an anti-stigma intervention

This study demonstrated that the VBCI is effective in improving attitudes of Penang primary care nurses towards people with mental illness. We found that this intervention was associated with immediate positive improvement in the attitudes of 30% of the nurses towards people with mental illness.

To our knowledge, this study is the first to evaluate the effectiveness of a short (4 minute-30 second) video as an anti-stigma intervention among a homogenous group of healthcare providers. It is also the first to utilise a Malay version of the OMS-HC-15 with Malaysian HCPs. Our findings are consistent with previous studies which supported the use of social contact as an effective anti-stigma intervention among HCPs [2, 26, 27, 46, 61–63]. Previous studies were conducted on a heterogenous group of HCPs [61–66], utilised videos which are much longer in duration (50 minutes) and included post-video discussions with participants [63, 66]. At the time of writing, there were no other published studies that had evaluated similar short videos as anti-stigma interventions in HCPs.

### Attitudes towards people with mental illness

In our study, the mean total and subscale scores (except for the ‘Help-seeking and Disclosure’ subscale) were found to be much higher than the mean scores from the study by Modgill and colleagues [48]. This implies that primary care nurses in Penang have more stigmatising attitudes towards people with mental illness compared to Canadian HCPs. Our figures were however lower than those found on a recent study on Malaysian medical students which utilised the same scale [67]. Taken together, it appears that significant stigmatising attitudes towards people with mental illness are prevalent among nurses and future doctors in Malaysia, a finding which is consistent with local and international studies [4, 29, 31, 34]. Anti-stigma interventions which target HCPs in all areas of healthcare provision, and especially those working in primary care should be carried out to improve mental health services.

### Factors associated with attitudes towards people with mental illness

In this study, attitudes towards people with mental illness (at baseline) were influenced by the nature of contact with people with mental illness and psychiatric training.

#### Nature of contact

Contrary to results from most studies [10, 12–15, 68], this study did not find any significant associations between the presence of prior contact with people with mental illness and the nurses’ attitudes towards them. However, our results were consistent with Couture and Penn’s [14] suggestions that there are other factors present during contact that can influence attitudes, rather than exposure to contact *per se*, such as the nature of contact (for example, a pleasant versus unpleasant interaction). Certainly, prejudice towards people with mental illness has been found in mental health professionals who have more contact with people with mental illness compared to the normal population [23, 28]. Again, this seems to suggest there are other factors apart from mere contact which influences attitude-forming [14, 69, 70] such as positive contact and psychiatric training, as we found in this study.

In this study, nurses with positive contact were less likely to be prejudiced towards people with mental illness in comparison to those who had neutral contact. The positive contact may have improved attitudes by reducing fear and increasing empathy towards people with mental illness. A meta-analysis has shown that the reduction of prejudice via contact may be mediated by factors such as reducing fear and anxiety towards people with mental illness, improving empathy with them, and increasing mental health literacy [71]. However, the meta-analysis found that the mediational value of providing evidence-based knowledge on mental illness has less positive effect compared to the other two factors.

Interestingly, the study did not find significant differences in stigmatising attitudes between those nurses who had reported positive, as compared to negative experiences of people with mental illness. This finding may be explained by the fact that only a small number of nurses reported experiences of negative contact (8%) compared to neutral contact (48%). Another possibility for this finding is that the nurses who classified their interactions as neutral were influenced by cultural beliefs which stigmatised mental illness [17, 29, 72]. For example, two studies found a tendency among the Malaysian public to perceive people with mental illness as dangerous, leading to fear and social distance [17, 72]. People with mental illness have also been regarded as bearers of bad luck, which contributes to more mental illness-related stigma [29]. Further studies are necessary to further explore cultural factors associated with stigmatising attitudes.

#### The influence of psychiatry training for nurses

In this study, the desire for and presence of any prior psychiatry training was associated with less stigmatising attitudes towards people with mental illness. The latter finding is consistent with the existing literature where greater mental health literacy has been correlated to less stigmatising attitudes [9, 18]. Nevertheless, the statistical significance for our finding in this regard is marginal (*p* = 0.049). Previous studies have reported that Malaysian nurses receive suboptimal training in best practices for provision of psychiatric care; many nurses do not receive appropriate training in psychiatry, and nurses who are working in psychiatry are not necessarily specialist-trained [73]. In addition, studies have shown that interventions which incorporate mental health education and contact are effective in reducing stigmatising attitudes towards people with mental illness [2, 27, 46, 74]. This suggests that the provision of on-going mental health training to supplement the existing knowledge may be useful in reducing stigmatising attitudes of nurses towards people with mental illness.

In our study, we found that the desire for psychiatric training correlated with less stigmatising attitudes towards people with mental illness. This finding has not been reported previously in any studies with HCPs. We suggest that this association may reflect a greater motivation in these nurses to obtain psychiatric training if it became available to them. However, the motivation to help people with mental illness is possibly deterred by factors such as a fear of people with mental illness, inadequate knowledge and uncertainty about how to manage and support people presenting with mental illness. This has been demonstrated in some studies in which fear and poor mental health literacy were identified as deterrents of effective caring of people with mental illness by HCPs [75–77]. Taken together, these findings highlight that primary care nurses require support in psychiatry-related education and training. Qualitative studies may be helpful in exploring these issues further.

### New insights

Finally, this study provided several new insights into the attitudes of Malaysian primary care nurses towards people with mental illness. Firstly, it is surprising to note that as many as 10% of the participants reported they had never encountered people with mental illness despite being frontline staff in primary care clinics, and given that the recent 2015 Malaysian National Health and Morbidity Survey reported that approximately 1 in 3 Malaysians aged 16 years and above suffer from some mental health condition at some point in time [47]. Moreover, both local and international studies have demonstrated that mental health problems are common amongst primary care attendees, with 20% to 50% suffering from at least one type of mental disorder [78–81]. One possible explanation for this finding is a lack of awareness and knowledge about mental health conditions, which has led to poor recognition of mental health problems by primary care nurses in people with mental illness [9, 82].

Secondly, less than a third of the participants indicated that they had any previous training. This is rather surprising given that the official curriculum for Malaysian undergraduate nursing training stipulates that all nurses receive approximately five weeks of integrated mental health training [73]. It is possible that some participants who responded to this question assumed it referred to on-going mental health training. On the other hand, the nurses’ answers in this regard may reflect that they received little or no mental health related-training, a finding that has been reported previously in Malaysia as reported by Oranye and colleagues [73].

Thirdly, although studies indicate that nurses are at increased risk of suffering mental health problems compared to the general population [83–86], only 2 nurses reported receiving any treatment for personal mental health conditions. The reasons for this finding are unknown. However, the results may reflect poor levels of knowledge and awareness of mental health conditions leading to poorer recognition of such problems in nurses themselves [82]. Another possibility is that these nurses are actually aware of their problems but for whatever reasons did not report them, even when reassured that their disclosures during the study would remain confidential [25, 30]. As noted by Hanafiah and colleagues [30], people with mental illness may not disclose their mental health status for fear of discrimination or of losing their jobs. In addition, their help-seeking behaviour may be influenced by cultural beliefs, leading to the tendency to seek help from alternative sources rather than from official psychiatric services [16, 87]. For example, there is evidence that some Malaysian caregivers prefer to seek treatment from traditional healers as they believe that mental illnesses are linked to supernatural causes, and only turn to psychiatric services after such interventions have failed [16, 87].

### Limitations

The study had several limitations. Firstly, Hawthorne’s effect may be present. Measures were taken to limit this effect during the conduct of the study. For example, participants were reminded that their answers were anonymous and that this was an independent academic research unrelated to their employment agency. The interaction between the researcher and participants were also kept to a minimal level to reduce the chances of participants being biased in choosing their answers.

Secondly, the VBCI only maintained partial fidelity to the key recommendations of effective anti-stigma interventions (such that there was no focus on skills-training or speaking to the audience about stigma and people with mental illness) due to time and financial constraints. Thirdly, this study did not measure effectiveness of the VBCI as an anti-stigma intervention beyond the immediate-term due to time constraints. As such, we were unable to assess whether the improvements in attitudes would be sustained, or whether booster VBCIs would be necessary to maintain progress as recommended by the review by Thornicroft and colleagues [26]. Fourthly, this is not a full randomized controlled trial which limited the quality of the intervention. Finally, a factor analysis was not included in the psychometric evaluation of the OMS-HC-15-M.

### Strengths

This study has several methodological strengths. Firstly, our sample was selected via simple random sampling to ensure equal representativeness from the entire state of Penang. Secondly, the sample characteristics are consistent with those found in other Malaysian studies [31, 73], with narrow confidence intervals, improving the general applicability of the findings to similar populations of primary care nurses in this region. Thirdly, the VBCI incorporated most of the Canadian key recommendations for effective anti-stigma intervention [2] as well as stereotype disconfirmation as proposed by Alport’s Intergroup Contact Theory [88]. Finally, the quality of the VBCI was ensured by obtaining media technical expertise and content expert opinion.

## Conclusions

The results from this preliminary study have highlighted a number of noteworthy issues that have implications for policy makers. This is the first study to evaluate a short video contact-based intervention as an anti-stigma intervention among HCPs in Malaysia, as well as being the first to utilise the Malay translation of the OMS-HC-15. Our study has extended current knowledge by providing some insights into the perceptions of Penang primary care nurses towards people with mental illness. We found stigmatising attitudes to be quite prevalent amongst primary care nurses in Penang and that a short VBCI is effective in improving attitudes of these nurses towards people with mental illness at least in the immediate term. Positive contact and psychiatric training were associated with less prejudice towards people with mental illness, which highlights the need for adequate contact-based psychiatric training among nurses. This is important given that most encounter people with mental illness, serve as frontline staff in health settings, and desire such training. In addition, less than a third (of our sample) stated that they received psychiatric training. We suggest an urgent need to incorporate on-going psychiatry-related training for these HCPs to help improve patient care and outcomes.

## Recommendations for future research and practice

Based on these results, several recommendations can be made. Our main recommendation is that primary care nurses should receive adequate evidence-based education on mental illnesses, and on-going psychiatry-related contact-based training in the assessment and management of people presenting with mental health problems.

We suggest that future research should utilise randomised controlled trial designs to account for potential confounding effects, limit a possible Hawthorne effect, and allow for direct comparison between control-recipients and intervention-recipients. In addition, the Canadian recommendations should be implemented in full by incorporating elements of skills-training and using passionate speakers in contact-based educational interventions. Also, we agree with the suggestion by Gronholm and colleagues that potential mediators of contact-prejudice should be taken into account, including improving mental health literacy, reducing fear and enhancing empathy towards people with mental illness [89].

We recommend that studies should be extended beyond a month’s duration with a longer follow-up to evaluate the effectiveness on a medium to long term period. Qualitative studies may be helpful in exploring stigmatising attitudes, especially in terms of enhancing greater understanding of cultural influences on HCP perceptions. Such studies may also provide information leading to a greater understanding of the training needs of the HCPs. Further, studies that evaluate the full psychometric properties of the OMS-HC-15-M are required. Finally, the authors plan to share these findings and recommendations with policy makers at the health ministry.

## Supporting information

S1 Table Univariate analysis (t test) of independent variables associated with OMS-HC-15-M mean total scores at baseline. OMS-HC-15-M = Opening Minds Scale for Healthcare Providers– 15 items Malay version, PWMI = people with mental illness, MI = mental illness, CI = Confidence Interval; *p<0.05; **using t test with unequal variances.
(PDF)

S2 Table Univariate analysis (one-way ANOVA) of independent variables associated with OMS-HC-15-M mean total scores at baseline. CI = confidence interval, PWMI = people with mental illness, *p<0.05; **adjusted p<0.017 for pair comparison test, ^†^Bonferroni testing carried out only on those with significance values *p*<0.05.
(PDF)

S1 File Dataset for ANPMI study. This dataset file includes description of the dataset, description of the variables and the raw data of the study.
(XLSX)
