# Supplementary material for: Determining the effectiveness of a video-based contact intervention in improving attitudes of Penang primary care nurses towards people with mental illness
Source: PLoS One. 2017 Nov 13;12(11):e0187861. doi: 10.1371/journal.pone.0187861 (PMC5683645; doi:10.1371/journal.pone.0187861)
Supplement: S1 Table — OMS-HC-15-M = Opening Minds Scale for Healthcare Providers– 15 items Malay version, PWMI = people with mental illness, MI = mental illness, CI = Confidence Interval; *p<0.05; **using t test with unequal variances. (PDF) [file pone.0187861.s001.pdf]

Supporting Information

S1 Table. Univariate analysis (t test) of independent variables associated with OMS-HC-15-M mean total scores at baseline.

**Variable**		**Mean score (95% CI)**	**t value**	***p* value**
Total scale		40 (39 – 40)	-	-
Aged 40 and below	Yes	39 (39 – 40)	1.1	0.278
	No	41 (39 - 43)		
Gender	Man	38 (19 – 56)	-0.57	0.568
	Woman	40 (39 – 40)		
Married	No	40 (38 – 42)	0.32	0.746
	Yes	40 (39 – 40)		
Had past contact with PWMI	No	41 (38 -44)	1.1	0.269
	Yes	39 (39 – 40)		
Had past contact with family/close friends with MI	No	40 (39 – 41)	0.81	0.419
	Yes	38 (36 – 41)		
Had past contact with acquaintances with MI	No	40 (39 – 41)	0.89	0.376
	Yes	38 (35 – 41)		
Had past contact with patients with MI	No	41 (39 – 43)	**2.5**	**0.013****
	Yes	39 (38 – 40)		
Had past contact with PWMI outside workplace	No	39 (37 – 40)	-1.6	0.117
	Yes	40 (39 – 41)		
Received previous training	No	40 (39 – 41)	**2.5**	**0.014***
	Yes	38 (37 – 39)		
Desire to be trained	No	42 (40 – 45)	2.3	**0.030****
	Yes	39 (38 – 40)		

OMS-HC-15-M = Opening Minds Scale for Healthcare Providers – 15 items Malay version, PWMI = people with mental illness, MI = mental illness, CI = Confidence Interval

**p*<0.05; **using t test with unequal variances
