# Supplementary material for: Determining the effectiveness of a video-based contact intervention in improving attitudes of Penang primary care nurses towards people with mental illness
Source: PLoS One. 2017 Nov 13;12(11):e0187861. doi: 10.1371/journal.pone.0187861 (PMC5683645; doi:10.1371/journal.pone.0187861)
Supplement: S2 Table — CI = confidence interval, PWMI = people with mental illness, *p<0.05; **adjusted p<0.017 for pair comparison test, †Bonferroni testing carried out only on those with significance values p<0.05. (PDF) [file pone.0187861.s002.pdf]

**S2 Table. Univariate analysis (one-way ANOVA) of independent variables associated with OMS-HC-15-M mean total scores at baseline.**

| | Variable | Mean score (95% CI) | F value | *p* value | Bonferroni t (95% CI)[†] | *p* value | adjusted *p* value |
|---|---|---|---|---|---|---|---|
| Ethnicity | Malay | 40 (39 – 41) | 4.4 | **0.014*** | -1.5 (-11 - 2.5) | 0.38 | |
| | Chinese | 36 (34 – 37) | | | -2.6 (-8.7 - -0.29) | 0.032 | 0.096 |
| | Indian | 35 (31 – 39) | | | -0.04 (-8.1 - 7.8) | 1.00 | |
| Religion | Islam | 40 (39 – 41) | 2.6 | 0.053 | - | - | - |
| | Buddhism | 36 (35 – 38) | | | | | |
| | Hinduism | 35 (30 – 40) | | | | | |
| | Christianity | 38 (29 – 46) | | | | | |
| Highest under-graduate qualification | Certificate | 39 (38 – 41) | 0.31 | 0.735 | - | - | - |
| | Diploma | 40 (39 – 41) | | | | | |
| | Degree | 38 (34 – 42) | | | | | |
| Rank | Community | 40 (39 – 41) | 0.61 | 0.611 | - | - | - |
| | nurse | 40 (38 – 41) | | | | | |
| | Staff nurse | 40 (37 – 43) | | | | | |
| | Head nurse | 37 (32 – 41) | | | | | |
| | Matron | | | | | | |
| Nature of encounter with PWMI | Pleasant | 38 (36 – 39) | 6.0 | **0.003*** | -1.17 (-5.8 - 2.0) | 0.74 | |
| | Unpleasant | 40 (36- 43) | | | 3.38 (0.83 - 5.0) | **0.003*** | **0.009**** |
| | Neutral | 41 (40 – 42) | | | 0.64 (-2.9 - 4.9) | 1.00 | |
| Frequency of encounter with PWMI | Daily | 40 (37 – 43) | 1.6 | 0.167 | - | - | - |
| | Once a week | 38 (36 – 41) | | | | | |
| | Once a | 37 (36 – 39) | | | | | |
| | month | 40 (39 – 41) | | | | | |
| | Rarely | | | | | | |

CI = confidence interval, PWMI = people with mental illness,

**p*<0.05; **adjusted p<0.017 for pair comparison test,

[†]Bonferroni testing carried out only on those with significance values *p*<0.05
